# Synthetic Cathinones Induce Developmental Arrest, Reduce Reproductive Capacity, and Shorten Lifespan in the *C. elegans* Model

**DOI:** 10.3390/jox15010033

**Published:** 2025-02-18

**Authors:** Cristina Mendes, Daniela Maia, Ricardo Jorge Dinis-Oliveira, Fernando Remião, Renata Silva, Daniel José Barbosa

**Affiliations:** 1 Associate Laboratory i4HB—Institute for Health and Bioeconomy, University Institute of Health Sciences—CESPU, 4585-116 Gandra, Portugal; a33272@alunos.cespu.pt (C.M.); danielamaia26@outlook.pt (D.M.); ricardo.dinis@iucs.cespu.pt (R.J.D.-O.); 2 UCIBIO—Applied Molecular Biosciences Unit, Translational Toxicology Research Laboratory, University Institute of Health Sciences (1H-TOXRUN, IUCS-CESPU), 4585-116 Gandra, Portugal; 3 Department of Public Health and Forensic Sciences and Medical Education, Faculty of Medicine, University of Porto, 4200-319 Porto, Portugal; 4 FOREN—Forensic Science Experts, Dr. Mário Moutinho Avenue, No. 33-A, 1400-136 Lisbon, Portugal; 5 Associate Laboratory i4HB—Institute for Health and Bioeconomy, Faculty of Pharmacy, University of Porto, 4050-313 Porto, Portugal; remiao@ff.up.pt (F.R.); rsilva@ff.up.pt (R.S.); 6 UCIBIO—Applied Molecular Biosciences Unit, Laboratory of Toxicology, Department of Biological Sciences, Faculty of Pharmacy, Porto University, 4050-313 Porto, Portugal; 7 i3S—Instituto de Investigação e Inovação em Saúde, Universidade do Porto, 4200-135 Porto, Portugal

**Keywords:** synthetic cathinones, methylone, pentedrone, 4-methylethcathinone, developmental arrest, reproductive behavior, lifespan, *C. elegans*

## Abstract

Drug abuse presents a significant global health challenge as the illicit drug market progresses from classic drugs to a growing prevalence of New Psychoactive Substances (NPS), particularly synthetic cathinones, which, although illegal, are often falsely marketed as safe and legal alternatives. The rapid increase in the use of these drugs complicates the assessment of their safety and effects on human health. However, they pose unique toxicological concerns that remain largely uncharacterized. This study investigated the toxic effects of three synthetic cathinones, namely, methylone, pentedrone, and 4-methylethcathinone (4-MEC), using the model organism *C. elegans*. We assessed the impact of these substances on animal survival, development, reproductive behavior, and longevity. Our results showed that short-term exposure (24 h) to concentrations of 5.0 mM or higher significantly reduced animal survival rates, while prolonged exposure (72 h) led to more pronounced toxicity, significantly reducing survival rates at concentrations as low as 1.0 mM. Moreover, sublethal concentrations resulted in developmental arrest. Additionally, pentedrone impaired reproductive capacity, while 4-MEC significantly shortened *C. elegans* lifespan. These findings highlight the urgent need for further investigation into the implications of synthetic cathinone use on human health through in vivo models as their prevalence in the illicit drug market continues to rise.

## 1. Introduction

Substance use disorder (SUD) is a medical condition characterized by an inability to control the use of legal or illegal drugs, alcohol, or medications. It is a complex disorder in which substance use leads to significant health problems [1].

Over the past few decades, the consumption of drugs of abuse has become an emerging problem, prompting authorities to implement measures to limit their use. Consequently, the illicit drug market has shifted from classic drugs such as 3,4-methylenedioxymethamphetamine (MDMA), cocaine, and heroin to New Psychoactive Substances (NPS), which are synthetic or semi-synthetic derivatives of known drugs [2,3]. These NPS are often unregulated and falsely marketed as safe and legal alternatives to classic illicit drugs [4], creating a vicious cycle where NPS are continuously synthesized and introduced into the illicit drug market. This creates an additional layer of risk, as there is neither sufficient time nor capacity to assess the toxicity of newly released NPS, posing a significant risk to human health.

**Figure 1:**
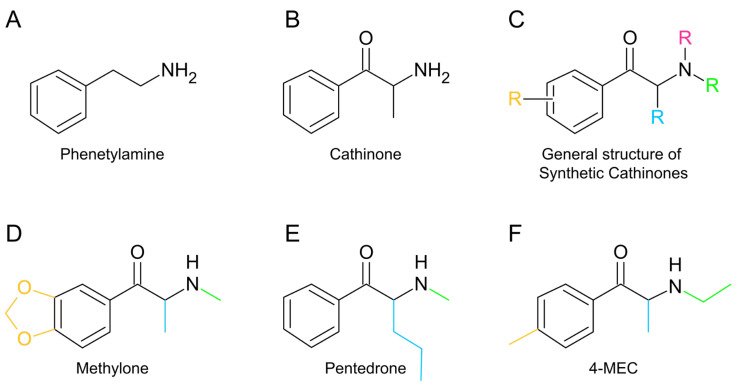
Chemical structures of phenethylamine, cathinone, and synthetic cathinones. (**A**,**B**) Chemical structures of phenethylamine (**A**), a natural central nervous system stimulant and neurotransmitter, and cathinone (**B**), which is naturally present in the Khat plant (*Catha edulis*). (**C**–**F**) General structure of synthetic cathinones (**C**) and the chemical structures of the synthetic cathinones used in this study: methylone (**D**), pentedrone (**E**), and 4-MEC (**F**). The chemical structures were drawn using ChemSketch Freeware 2023.2.4 [27].

Synthetic cathinones, a class of NPS also known as “bath salts”, are synthetic derivatives (Figure 1C) of the natural alkaloid cathinone (Figure 1B) found in the khat plant (*Catha edulis*) [5]. These substances are similar to the natural central nervous system (CNS) stimulant and neurotransmitter phenethylamine (Figure 1A). The introduction of substituent groups at the alpha carbon and other positions of the shared β-keto phenethylamine backbone (Figure 1C) generates a myriad of compounds, often with psychoactive effects more potent than those of classical drugs [6,7,8]. Their ease of synthesis and widespread availability via the internet or “smart shops” have contributed to their popularity [5]. Based on their effects on neurotransmitter systems, synthetic cathinones can be categorized into three distinct groups [2,3,6,9,10]. The first group includes synthetic cathinones like mephedrone (4-methylmethcathinone, 4-MMC), methylone (3,4-methylenedioxy-*N*-methylcathinone; Figure 1D), and ethylone (3,4-methylenedioxy-*N*-ethylcathinone), which produce effects similar to those of cocaine and MDMA by non-selectively inhibiting the reuptake of neurotransmitters and promoting the release of serotonin. The second group includes drugs such as pentedrone (Figure 1E), methcathinone, 4-fluoromethcathinone (4-FMC), 4-chloromethcathinone (4-CMC), and 4-methylethcathinone (4-MEC; Figure 1F), which show effects similar to those of methamphetamine by primarily inhibiting the reuptake transporters of dopamine (DAT) and noradrenaline (NAT), while promoting the release of dopamine. The last group includes pyrovalerone cathinones, which share a pyrrolidine structure and selectively inhibit DAT and NAT without affecting the release of monoamines. This group includes pyrovalerone and 3,4-methylenedioxypyrovalerone (MDPV), among others [2,3,6,9,10].

According to the European Union Drugs Agency (EUDA), the use of synthetic cathinones has been continuously rising. In 2022, a total of 26.5 tons of synthetic cathinones were seized in the European Union, compared to 3.3 tons in 2020 and 4.5 tons in 2021, which supports their accelerated growth in the illicit drug market [11,12], thereby raising significant health problems.

Synthetic cathinones induce agitation, delusions, hallucinations, hypertension, tachycardia, and hyperthermia, which likely contribute to their toxicity [3,6,7,13]. Studies have shown neurotoxicity associated with these drugs through mechanisms involving intracellular oxidative stress, mitochondrial dysfunction, and apoptosis [10,14,15,16,17,18,19,20,21,22,23]. However, most of these studies have been conducted in vitro using two-dimensional models [14,15,16,17,18,19,20,21,22], limiting human relevance. Thus, reports on both fatal and non-fatal cases of synthetic cathinone intoxication have been crucial in providing key insights into their toxic effects on humans [23,24,25,26]. The use of in vivo models to study the toxicity of these substances is thus essential, as they preserve the complex systemic interactions present in living organisms and are more representative of the human scenario. Additionally, in vivo models are better suited for assessing the effects of these substances on key aspects of human health, such as development, reproductive behavior, and longevity, which cannot be adequately assessed using in vitro models and remain largely unknown.

*Caenorhabditis elegans* (*C. elegans*) is a small, transparent hermaphrodite nematode with a simple multicellular structure that allows for easy observation of developmental processes and physiological changes under a microscope [28]. Its small size, rapid life cycle (approximately 3.5 days) that progresses through four larval stages (L1 to L4), from the embryonic stage to adulthood, and ease of cultivation make it well-suited for high-throughput experiments [29,30]. Also, its short lifespan (approximately 3 weeks) and rapid reproductive cycle [28] make *C. elegans* a suitable model for studying the influence of substances on development, reproductive behavior, and longevity. Moreover, the animal has a fully sequenced genome, with about 60–80% of human genes having a corresponding ortholog in *C. elegans* [31]. Additionally, certain aspects of cellular functioning, as well as neuronal and regulatory mechanisms, are conserved between *C. elegans* and humans [32], and it possesses most of the primary tissues found in more advanced multicellular organisms. Thus, findings from this animal model can be relevant to higher organisms, including humans.

This study aimed at evaluating the toxic effects of synthetic cathinones in *C. elegans*. By assessing the impact of these drugs on animal development, reproductive behavior, and lifespan, this research provides valuable insights into their safety and potential health risks for humans. We show that short-term (24 h) or prolonged (72 h) exposure to synthetic cathinones methylone, pentedrone, and 4-MEC led to a concentration-dependent reduction in animal survival and that sublethal concentrations caused developmental arrest. Additionally, pentedrone reduced the reproductive capacity of the animals, while 4-MEC shortened their lifespan. These findings not only highlight the specific toxic effects of synthetic cathinones in a model organism but also emphasize the broader implications for human health.

## 2. Materials and Methods

### 2.1. Synthetic Cathinones

Synthetic cathinones, methylone, pentedrone, and 4-MEC, were generously provided by the Portuguese Judiciary Police. One-dimensional (1D) ^1^H-Nuclear Magnetic Resonance (^1^H-NMR) spectroscopy was performed to assess their purity. For this purpose, on the day of the 1D ^1^H-NMR analysis, drug solutions at a concentration of 5.0 mM were prepared in deuterium oxide (D_2_O) with 1 mM 3-(trimethylsilyl)propionic-2,2,3,3-d4 acid (TSP), vortexed, and 600 µL were transferred to 5 mm NMR tubes. 1D ^1^H-NMR spectroscopy was conducted at the “Nuclear Magnetic Resonance Laboratory of the Materials Centre”, University of Porto (CEMUP), and the analysis was performed on a Bruker Avance III 400 9.4 T spectrometer (Bruker BioSpin, Rheinstetten, Germany) operating at 400 MHz (298 K) using the zg30 pulse sequence (Bruker library). The obtained 1D ^1^H-NMR spectra are depicted in Figure S1. In silico prediction of their lipophilicity (Consensus Log *P*_o/w_) and skin permeation (Log *K*_p_) was performed with the SwissADME free web tool [27].

### 2.2. C. elegans Maintenance

The *C. elegans* strain DC19 [*bus-5(br19)*] was used in this study. Animals of this strain exhibit increased permeability of the outer cuticle, which makes them particularly suitable for evaluating the effects of exogenous compounds, including drugs of abuse [33]. The strain was maintained at 22 °C on standard Nematode Growth Medium (NGM) plates seeded with *E. coli* OP50 bacteria.

For preparing NGM plates, 3 g of NaCl (Ref: 1064041000, Merck, Darmstadt, Germany), 2.5 g of Tryptone/Peptone (Ref: 8952.3, Carl Roth, Karlsruhe, Germany), and 17 g of agar (Ref: MB02903, NZYtech, Lisbon, Portugal) were dissolved in 975 mL of ddH_2_O (final volume of 1000 mL), and the medium was autoclaved at 110 °C for 30 min. After cooling to 50 °C, the medium was supplemented with 1 mM CaCl_2_.2H_2_O (Ref: 223506, Merck, Darmstadt, Germany; 1 M stock solution prepared in ddH_2_O and autoclaved), 1 mM MgSO_4_.7H_2_O, (Ref: 230391, Merck, Darmstadt, Germany; 1 M stock solution prepared in ddH*_2_*O and autoclaved), 1 mM KH_2_PO_4_ (Ref: P9791, Merck, Darmstadt, Germany; 1 M stock solution prepared in ddH_2_O, adjusted to pH 6, and autoclaved), and 5 µg/mL cholesterol (Ref: C8667, Sigma-Aldrich, Darmstadt, Germany; 5 mg/mL stock solution prepared in absolute ethanol). The medium was manually dispensed into 35 mm diameter plastic plates (Ref: BP53-06, Corning, New York, NY, USA) at an 8 mL/plate ratio and left to dry at room temperature for 7 days. After this period, 250 µL of OP50 bacterial suspension was added to the plates and left to dry at room temperature for 2 additional days. Following this, the plates were ready for use.

### 2.3. Synchronization of Animals at the L1 Stage

All experiments started with animals synchronized at the L1 stage. Seven days before synchronization, L1 animals were allowed to grow on OP50-seeded plates (6 animals per plate; 8 plates) until the day of synchronization (plates were full of gravid adults). Adult animals were collected by washing the plates with M9 buffer [86 mM NaCl, 42 mM Na_2_HPO_4_ (Ref: S3264, Merck, Darmstadt, Germany), 22 mM KH_2_PO_4_, 1 mM MgSO_4_.7H_2_O], polled together in a 15-mL falcon tube and pelleted via centrifugation at 800× *g* for 3 min at room temperature. After 2 additional washes with 10 mL of M9, 5 mL of bleaching solution (67% M9, 22% household bleach, 11% 5 N NaOH) was added, and the worm suspension was vortexed for 4 min. Embryos were pelleted at 800× *g*, for 3 min, washed 3× with 10 mL M9 buffer, and allowed to develop and hatch in M9 buffer overnight at room temperature with shacking (150 rpm).

### 2.4. Exposure to Synthetic Cathinones

Synchronized L1s (~200/condition) were exposed, in 48-well plates, to growing concentrations of synthetic cathinones and allowed to incubate for 24 h (short-term exposure) or 72 h (prolonged exposure) in M9 buffer containing OP50 bacteria as a food source. All incubations were performed at a room temperature of 22 °C. Importantly, during the incubation period, no drug replenishment was performed; only a single exposure was applied at the beginning of the experiments. After drug exposure, animals were transferred to OP50-seeded NGM plates and processed for different experiments. Stock solutions of synthetic cathinones (50 mM) were prepared in M9 buffer and stored at −20 °C.

### 2.5. Animal Survival Test

The effects of synthetic cathinones on animal survival were evaluated by exposing synchronized L1-stage animals to increasing concentrations of synthetic cathinones and allowed to incubate for 24 h (0.0, 0.1, 0.5, 1.0, 5.0, 7.5, or 10.0 mM) or 72 h (0.0, 0.5, 1.0, 2.5, or 5.0 mM). The animals were then transferred to drug-free OP50-seeded NGM plates and allowed to acclimate for 30 min to an hour (to ensure sufficient time for potentially reversible effects, such as anesthesia, to disappear), and the number of surviving and dead worms was counted using a stereoscope. Animals were scored as dead if they did not move and if there was no evidence of pharyngeal pumping. Since animals were only monitored for movement and pharyngeal pumping after the acclimation period, we were able to more accurately distinguish between temporary immobilization and true mortality. Results are expressed as the percentage of surviving animals. Drugs were tested in 5 to 11 independent experiments conducted on different days.

### 2.6. Animal Development Assay

The effects of synthetic cathinones on animal development were evaluated by exposing synchronized L1-stage animals to different concentrations of synthetic cathinones (0.0, 0.5, or 1.0 mM) and allowed to incubate for 72 h. Then, the animals were transferred to OP50-seeded NGM plates. Live animals (~20 in each independent experiment) were imaged at room temperature using an SMZ 745T stereoscope (Nikon, Tokyo, Japan) equipped with an MD-E3ISPM-E3 8.3 Touptek complementary metal oxide semiconductor (CMOS) camera (AmScope, Irvine, CA, USA) and controlled using ToupView software (version x64, 4.11.19728.20211022, ToupTek Photonics, Hangzhou, China). Measurements of animal length were performed using Fiji software ((Image J version 2.14.0/1.54F) [34] by tracing a segmented line along the animal’s body. Results are expressed as the length of the animals relative to the control condition (0.0 mM). Drugs were tested in 4 to 5 independent experiments conducted on different days.

### 2.7. Brood Size and Embryonic Viability Tests

The effects of synthetic cathinones on brood size and embryonic viability were assessed by exposing synchronized L1-stage animals (F0) to different concentrations of synthetic cathinones (0.0, 0.5, or 1.0 mM) and allowed to incubate for 72 h. Then, the animals (F0 generation) were transferred to OP50-seeded NGM plates. Live animals (F0), at the same development stage, were singled out to new OP50-seeded NGM plates and allowed to lay embryos (F1 generation) for 72 h. After that period, mothers (F0) were removed, and 24 h later, the number of larvae and unhatched embryos (F1) was counted. Brood size results are expressed as the total number of progeny, whereas F1 embryonic viability is expressed as the percentage of hatched embryos. Drugs were tested in 3 to 7 independent experiments conducted on different days.

### 2.8. Lifespan Assay

The effects of synthetic cathinones on animal lifespan were investigated by exposing synchronized L1-stage animals to different concentrations of synthetic cathinones (0.0, 0.5, or 1.0 mM) and allowed to incubate for 72 h. Then, live animals were transferred to OP50-seeded NGM plates and examined every 2–3 days throughout their lifespan. When necessary, the animals were transferred to new OP50-seeded NGM plates to remove the progeny. Animals were considered dead if they did not respond to gentle touch or showed no evidence of pharyngeal pumping. Animals that escaped or were found dead at the edge of the plate were excluded from the assay. Results are expressed as the percentage of surviving animals over time. Drugs were tested in 3 to 6 independent experiments conducted on different days.

### 2.9. Statistical Analysis

The software GraphPad Prism 8.0.2 for Windows (GraphPad Software, Inc., San Diego, CA, USA) was used for statistical analysis of the data. Values in the figures represent the mean ± standard error of the mean (SEM) from the number of independent experiments indicated in the figure legends. The normality of the data was assessed using four tests: Anderson–Darling, Kolmogorov–Smirnov, D’Agostino and Pearson omnibus, and Shapiro–Wilk normality tests. Statistical differences among groups were assessed using the Kruskal–Wallis nonparametric test, followed by Dunn’s multiple comparison test. For lifespan analysis, the Log-rank (Mantel–Cox) test was used. Significant differences were set at *p* values below 0.05, with * *p <* 0.05, ** *p* < 0.01, *** *p* < 0.001, and **** *p* < 0.0001. Details of the statistical analysis performed are provided in the figure legends.

## 3. Results

### 3.1. Short-Term Exposure to the Synthetic Cathinones Methylone, Pentedrone, and 4-MEC Led to a Concentration-Dependent Reduction in Animal Survival

We used 1D ^1^H-NMR to confirm the purity of the synthetic cathinones. As shown in Figure S1, the compounds exhibited a high degree of purity, indicating their suitability for laboratory testing.

**Figure 2:**
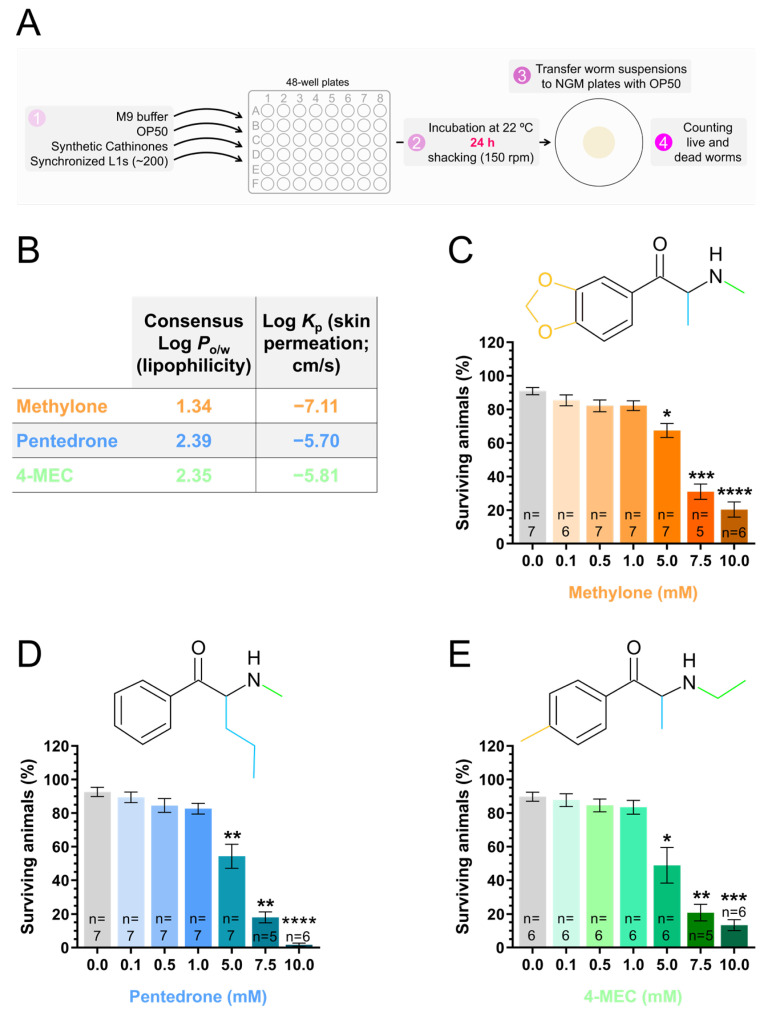
Short-term exposure (24 h) to the synthetic cathinones methylone, pentedrone, and 4-MEC led to a concentration-dependent reduction in animal survival: (**A**) Schematic representation of the short-term incubation protocol and animal survival assessment. (**B**) In silico prediction of lipophilicity (Consensus Log Po/w) and skin permeation (Log Kp) for the synthetic cathinones used in this study, performed with the SwissADME free web tool. (**C**–**E**) Survival rates of animals exposed to increasing concentrations (0.0, 0.1, 0.5, 1.0, 5.0, 7.5, or 10.0 mM) of methylone (**B**), pentedrone (**C**), or 4-MEC (**D**), from the L1 stage, for 24 h. The mean ± SEM represents the percentage of surviving animals from 5 to 7 independent experiments (n) performed on different days. Statistical differences were analyzed using the Kruskal–Wallis nonparametric test, followed by Dunn’s multiple comparison test [* *p* < 0.05; ** *p* < 0.01; *** *p* < 0.001; **** *p* < 0.0001 versus 0.0 mM (control)].

The recreational use of synthetic cathinones follows distinct patterns. While some users take these drugs sporadically or for short periods, others use them regularly over extended durations [35]. Based on this rationale, we initially studied the potentially toxic effects of exposing synchronized L1-stage animals to increasing concentrations (0.0, 0.1, 0.5, 1.0, 5.0, 7.5, or 10.0 mM) of the synthetic cathinones methylone, pentedrone, and 4-MEC for 24 h (short-term exposure) by evaluating the animal survival rate (Figure 2A). For concentrations up to 1.0 mM, survival rates did not significantly differ from the control [percentage of surviving animals: (1) methylone: 0.0 mM (control) = 90.9 ± 2.2%; 0.1 mM = 85.4 ± 3.2% (*p* > 0.05); 0.5 mM = 82.1 ± 3.5% (*p* > 0.05); 1.0 mM = 82.2 ± 2.9% (*p* > 0.05); (2) pentedrone: 0.0 mM (control) = 92.6 ± 2.7%; 0.1 mM = 89.4 ± 3.2% (*p* > 0.05); 0.5 mM = 84.5 ± 4.1% (*p* > 0.05); 1.0 mM = 82.6 ± 3.2% (*p* > 0.05); (3) 4-MEC: 0.0 mM (control) = 89.8 ± 2.7%; 0.1 mM = 87.7 ± 3.8% (*p* > 0.05); 0.5 mM = 84.6 ± 3.8% (*p* > 0.05); 1.0 mM = 83.4 ± 4.1% (*p* > 0.05)] (Figure 2C–E; Table S1). However, exposure to synthetic cathinones at concentrations of 5.0 mM, 7.5 mM, and 10.0 mM significantly reduced the animal survival rate [percentage of surviving animals: (1) methylone: 0.0 mM (control) = 90.9 ± 2.2%; 5.0 mM = 67.4 ± 4.1% (*p* < 0.05); 7.5 mM = 30.9 ± 4.6% (*p* < 0.001); 10.0 mM = 20.3 ± 4.5% (*p* < 0.0001); (2) pentedrone: 0.0 mM (control) = 92.6 ± 2.7%; 5.0 mM = 54.3 ± 7.2% (*p* < 0.01); 7.5 mM = 18.0 ± 3.3% (*p* < 0.01); 10.0 mM = 1.7 ± 1.0% (*p* < 0.0001); (3) 4-MEC: 0.0 mM (control) = 89.8 ± 2.7%; 5.0 mM = 48.9 ± 10.6% (*p* < 0.05); 7.5 mM = 20.8 ± 4.9% (*p* < 0.01); 10.0 mM = 13.3 ± 3.3% (*p* < 0.0001)] (Figure 2C–E; Table S1). These results demonstrated that short-term exposure (24 h) to synthetic cathinones at concentrations of 5 mM or higher resulted in highly pronounced toxic effects in the *C. elegans* animal model. Among the three synthetic cathinones studied, methylone showed the lowest toxicity potential. This was consistent with its lower lipophilicity and skin permeation characteristics predicted in silico, compared to pentedrone and 4-MEC (Figure 2B).

### 3.2. Long-Term Exposure to the Synthetic Cathinones Methylone, Pentedrone, and 4-MEC Led to a Concentration-Dependent Reduction in Animal Survival

**Figure 3:**
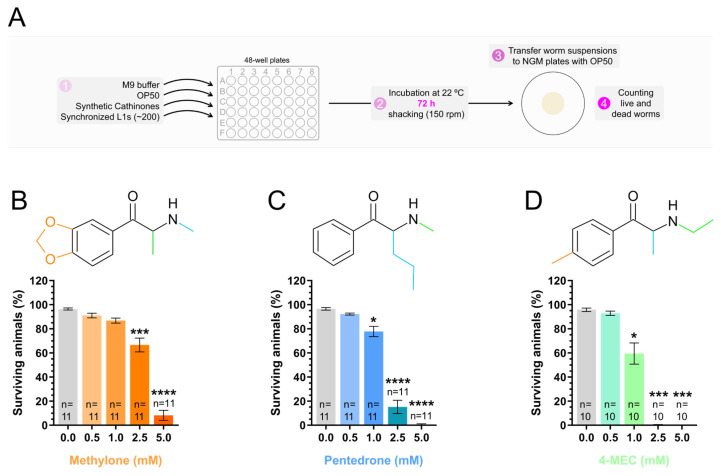
Long-term exposure (72 h) to the synthetic cathinones methylone, pentedrone, and 4-MEC led to a concentration-dependent reduction in animal survival. (**A**) Schematic representation of the long-term incubation protocol and animal survival assessment. (**B**–**D**) Survival rates of animals exposed to increasing concentrations (0.0, 0.1, 0.5, 1.0, 5.0, 7.5, or 10.0 mM) of methylone (**B**), pentedrone (**C**), or 4-MEC (**D**), from the L1 stage, for 72 h. The mean ± SEM represents the percentage of surviving animals from 10 to 11 independent experiments (n) performed on different days. Statistical differences were analyzed using the Kruskal–Wallis nonparametric test, followed by Dunn’s multiple comparison test [* *p* < 0.05; *** *p* < 0.001; **** *p* < 0.0001 versus 0.0 mM (control)].

After assessing the short-term toxic effects of synthetic cathinones on animal survival, we investigated the impact of prolonged exposure (Figure 3A). To this end, synchronized L1-stage animals were exposed to increasing concentrations of synthetic cathinones (0.0, 0.5, 1.0, 2.5, or 5.0 mM) and incubated for 72 h. Exposure to 0.5 mM of the three synthetic cathinones, or 1.0 mM of methylone for 72 h, did not significantly alter the survival rate of the animals compared to the control [percentage of surviving animals: (1) methylone: 0.0 mM (control) = 96.4 ± 0.9%; 0.5 mM = 91.0 ± 2.0% (*p* > 0.05); 1.0 mM = 86.8 ± 2.1% (*p* > 0.05); (2) pentedrone: 0.0 mM (control) = 96.5 ± 1.1%; 0.5 mM = 92.2 ± 0.8% (*p* > 0.05); (3) 4-MEC: 0.0 mM (control) = 95.8 ± 1.5%; 0.5 mM = 92.9 ± 1.8% (*p* > 0.05)] (Figure 3B–D; Table S2). However, exposure to 1.0, 2.5 and 5.0 mM of pentedrone or 4-MEC, as well as 2.5 and 5.0 mM of methylone, significantly reduced animal survival compared to the control [percentage of surviving animals: (1) methylone: 0.0 mM (control) = 96.4 ± 0.9%; 2.5 mM = 66.6 ± 5.8% (*p* < 0.001); 5.0 mM = 8.2 ± 4.2% (*p* < 0.0001); (2) pentedrone: 0.0 mM (control) = 96.5 ± 1.1%; 1.0 mM = 77.8 ± 4.3% (*p* < 0.05); 2.5 mM = 15.2 ± 5.5% (*p* < 0.0001); 5.0 mM = 0.6 ± 0.6% (*p* < 0.0001); (3) 4-MEC: 0.0 mM (control) = 95.8 ± 1.5%; 1.0 mM = 59.4 ± 8.8% (*p* < 0.05); 2.5 mM = 0.3 ± 0.2% (*p* < 0.001); 5.0 mM = 0.0 ± 0.0% (*p* < 0.001)] (Figure 3B–D; Table S2).

These results indicated that prolonged exposure (72 h) of *C. elegans* to synthetic cathinones at 2.5 mM and 5.0 mM induced severe toxic effects, significantly intensifying their toxicity compared to short-term exposure (24 h). Importantly, since the drugs were not replenished during the incubation period, this result suggests that they likely persisted in the medium over time, allowing for continued exposure and uncovering more extensive toxic effects that may not have been evident within the initial 24 h. Additionally, the lower toxicity of methylone under prolonged exposure, compared to pentedrone and 4-MEC, was consistent with the results observed after 24 h of exposure and with the in silico predictions of its lipophilicity and skin permeation characteristics (Figure 2B–E).

### 3.3. Sublethal Concentrations of Synthetic Cathinones Arrested Animal Development

The increasing use of drugs among adolescents and young adults is a major health concern, as their brain and body are still developing [36,37]. Similar concern exists regarding drug use during pregnancy due to the potential effects on fetal development [38,39].

**Figure 4:**
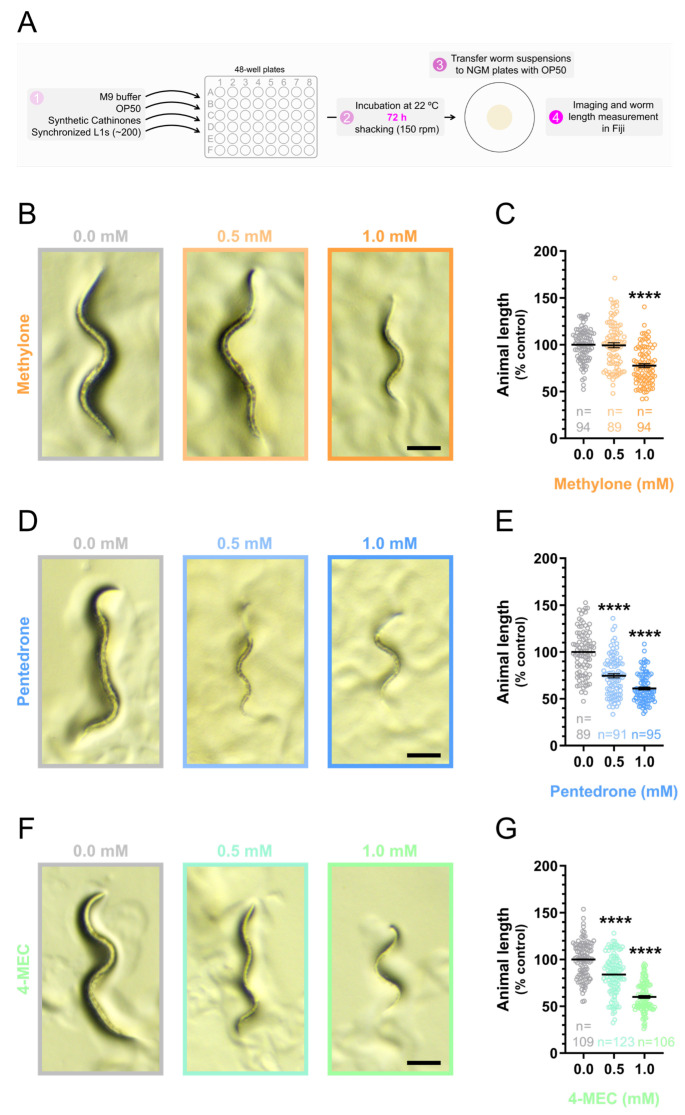
Sublethal concentrations of synthetic cathinones arrested animal development. (**A**) Schematic representation of the protocol used to assess the effect of synthetic cathinones on *C. elegans* development after a prolonged incubation period of 72 h. (**B**,**D**,**F**) Representative brightfield images of animals exposed to the synthetic cathinones methylone (**B**), pentedrone (**D**), or 4-MEC (**F**) at concentrations of 0.5 mM or 1.0 mM from the L1 stage, for 72 h. Scale bar: 100 µm. (**C**,**E**,**G**) Length of animals exposed to the synthetic cathinones methylone (**C**), pentedrone (**E**), or 4-MEC (**G**) at concentrations of 0.5 or 1.0 mM, from the L1 stage, for 72 h. Animal length (% of control) is represented as the mean ± SEM of the indicated number of animals (n), determined in 4 to 5 independent experiments performed on different days. Statistical differences were analyzed with the Kruskal–Wallis nonparametric test, followed by Dunn’s multiple comparison test [**** *p* < 0.0001 versus 0.0 mM (control)].

The life cycle of *C. elegans* progresses through four larval stages (L1 to L4), from the embryonic stage to adulthood. Each larval stage is characterized by specific developmental processes, including growth, organ development, and the establishment of neural and muscular systems. Additionally, the entire life cycle of the organism typically takes about 3.5 days at optimal growth temperatures [30,40]. This makes *C. elegans* a powerful model for studying the influence of substances on development. Based on this, we further explored the influence of synthetic cathinones (0.5 mM and 1.0 mM for 72 h) on *C. elegans* development (Figure 4A). The prolonged exposure protocol was chosen as it allows for testing lower concentrations of the drugs and effectively mimics the extended periods of synthetic cathinone consumption that have been documented [41,42].

*C. elegans* development can be assessed in several ways: morphological analysis (measuring body length or identifying developmental stages) [43], life cycle timing (e.g.*,* time from embryo to adulthood) [44], reproductive output (brood size) [45], or gene expression analysis [46]. Compared to other methods, measuring the animal’s body length is quicker and can be performed earlier in the life cycle than counting brood size (which requires animals to be in adulthood). Moreover, this method does not require tracking the animals over a specific period, as it is necessary for determining life cycle timing, and it does not require advanced techniques like gene expression analysis. Additionally, body length correlates well with the developmental stage [43] and can be easily applied to measure large numbers of animals without requiring sophisticated or expensive equipment. Therefore, it represents a straightforward and effective alternative for evaluating the impact of substances on *C. elegans* development in toxicological studies. Consequently, in this study, the potential influence of synthetic cathinones on development was estimated by measuring the animal’s body length (Figure 4A).

Exposure to a sublethal concentration of 0.5 mM of pentedrone (Figure 4D,E; Table S3) or 4-MEC (Figure 4F,G; Table S3) significantly reduced animal length [% of control: pentedrone = 74.6 ± 2.2% (*p* < 0.0001); 4-MEC = 84.0 ± 1.9% (*p* < 0.0001)]. Additionally, at a concentration of 1 mM (sublethal concentration of methylone), the three synthetic cathinones under study produced even more pronounced effects on animal development [animal length (% control): methylone = 77.6 ± 2.1% (*p* < 0.0001); pentedrone = 61.1 ± 1.6% (*p* < 0.0001); 4-MEC = 60.0 ± 1.5% (*p* < 0.0001)] (Figure 4B–G; Table S3). These results indicated that the synthetic cathinones, at sublethal concentrations, interfered with the normal growth and developmental processes of the organism.

### 3.4. Pentedrone Impaired C. elegans Reproductive Behavior

**Figure 5:**
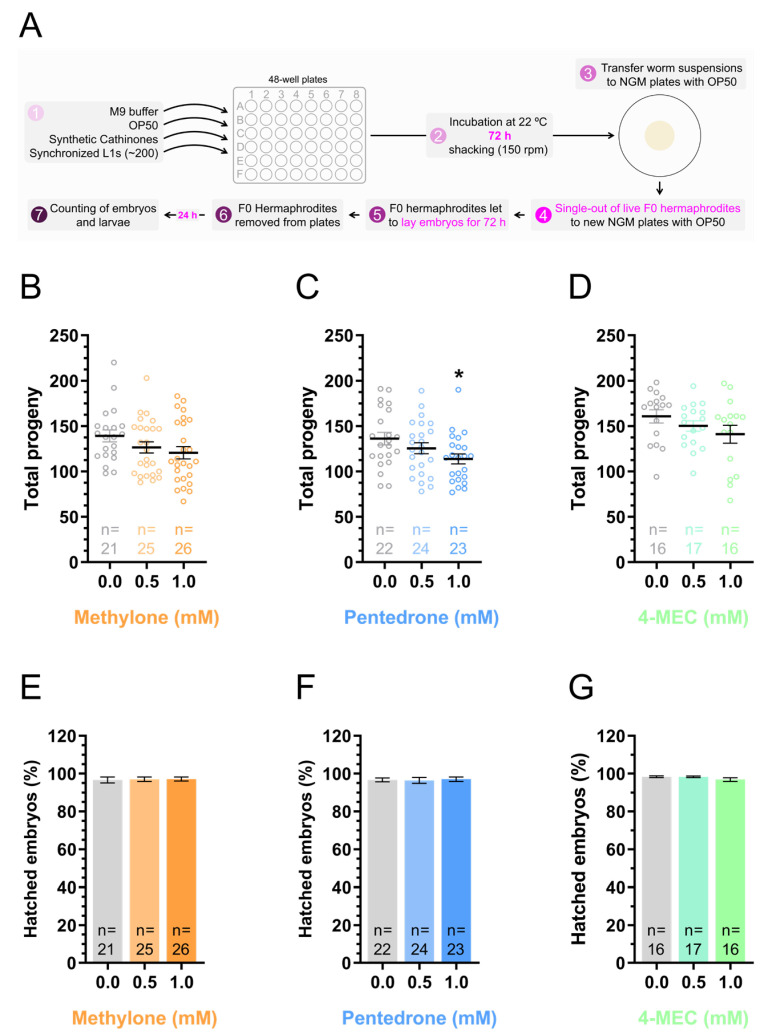
Pentedrone reduced the *C. elegans* brood size. (**A**) Schematic representation of the protocol used to assess the consequences of synthetic cathinones on *C. elegans* reproductive behavior after an incubation period of 72 h. (**B**–**D**) Brood size of animals exposed to the synthetic cathinones methylone (**B**), pentedrone (**C**), or 4-MEC (**D**) at concentrations of 0.5 or 1.0 mM, from the L1 stage, for 72 h. Data are represented as the mean ± SEM of the total progeny from the indicated number of F0 animals (n), assessed in 3 to 6 independent experiments performed on different days. Statistical differences were analyzed using the Kruskal–Wallis nonparametric test, followed by Dunn’s multiple comparison test [* *p* < 0.05 versus 0.0 mM (control)]. (**E**–**G**) Viability of the progeny (F1 generation) from animals exposed to the synthetic cathinones methylone (**E**), pentedrone (**F**), or 4-MEC (**G**) at concentrations of 0.5 or 1.0 mM, from the L1 stage, for 72 h. The percentage of hatched embryos (F1 generation) is represented as the mean ± SEM from the indicated number of F0 animals (n), determined in 3 to 7 independent experiments performed on different days. Statistical analysis was performed using the Kruskal–Wallis nonparametric test.

Various studies indicate that drugs of abuse can influence reproductive behavior in both humans and animals. Several substances, including ethanol, tobacco, cannabis, opioids, and CNS stimulants, are associated with changes in sexual function, fertility, and reproductive performance [39,47]. The short lifespan and rapid reproductive cycle of *C. elegans* [48] make it a powerful model for studying the interference of substances or environmental influences on reproduction. Based on this, we further explored the potential consequences of synthetic cathinones (72 h of exposure) in *C. elegans* reproductive behavior by evaluating their brood size (each animal was individually assessed; Figure 5A). For the different experimental conditions analyzed (0.0, 0.5, and 1.0 mM), animals at the same developmental stage were selected to eliminate possible interference from developmental differences between experimental conditions.

Exposure to methylone and 4-MEC at both tested concentrations, or to 0.5 mM pentedrone, did not significantly alter the animal brood size [(1) methylone: 0.0 mM (control) = 139.2 ± 6.5; 0.5 mM = 126.6 ± 6.1 (*p >* 0.05); 1 mM = 120.6 ± 6.8 (*p >* 0.05); (2) pentedrone: 0.0 mM (control) = 136.2 ± 6.9; 0.5 mM = 125.5 ± 6.1 (*p >* 0.05); (3) 4-MEC: 0.0 mM (control) = 160.7 ± 7.2; 0.5 mM = 150.1 ± 5.9 (*p >* 0.05); 1 mM = 141.0 ± 9.8 (*p >* 0.05)] (Figure 5B–D; Table S4). However, animals exposed to 1.0 mM of pentedrone showed a significant reduction in brood size [0.0 mM (control) = 136.2 ± 6.9; 1.0 mM = 113.8 ± 5.6 (*p* < 0.05)] (Figure 5C; Table S4). This result highlighted the potentially detrimental effects of pentedrone on fertility and underscored the need for further investigation into its impact on reproductive health. These findings could also be particularly relevant for more potent synthetic cathinones currently available in the illicit drug market.

We extended our analysis beyond brood size and evaluated whether synthetic cathinones could affect the viability of the progeny (F1 generation) of animals exposed to the drugs (F0 generation) (Figure 5A). F1 viability was estimated by evaluating the percentage of embryos released by each animal, individually, that hatched (viable) in relation to the total number of embryos released (viable and non-viable). None of the tested drugs caused significant changes in the percentage of hatched F1 embryos [(1) methylone: 0.0 mM (control) = 96.7 ± 1.6%; 0.5 mM = 97.0 ± 1.2% (*p* > 0.05); 1 mM = 97.1 ± 1.1 (*p* > 0.05); (2) pentedrone: 0.0 mM (control) = 96.7 ± 1.0%; 0.5 mM = 96.4 ± 1.6% (*p* > 0.05); 1 mM = 97.1 ± 1.2% (*p* > 0.05); (3) 4-MEC: 0.0 mM (control) = 98.3 ± 0.5%; 0.5 mM = 98.3 ± 0.4% (*p* > 0.05); 1 mM = 96.7 ± 1.0% (*p* > 0.05)] (Figure 5E–G; Table S5). This result indicated that the exposure of animals to sublethal concentrations of synthetic cathinones did not alter the viability of their progeny.

### 3.5. Exposure to 4-MEC Shortened the Lifespan of C. elegans

**Figure 6:**
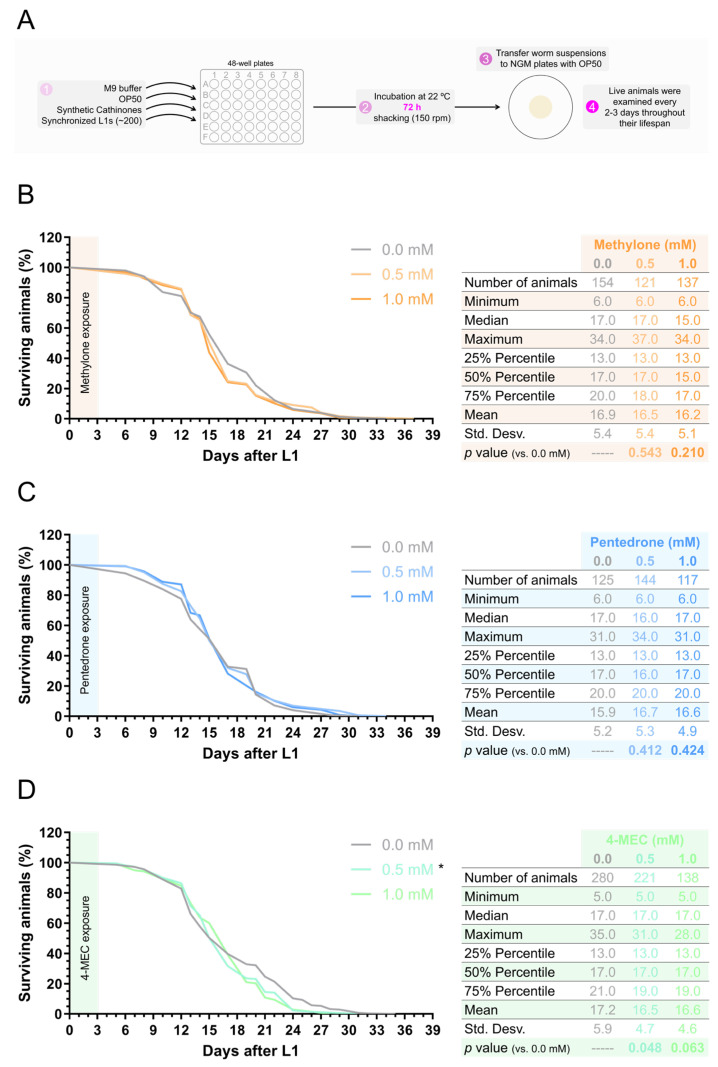
The synthetic cathinone 4-MEC reduced the lifespan of *C. elegans*. (**A**) Schematic representation of the protocol used to assess the influence of synthetic cathinones on *C. elegans* lifespan after a long-term incubation period of 72 h. (**B**–**D**) Lifespan curves of animals exposed to the synthetic cathinones methylone (**B**), pentedrone (**C**), or 4-MEC (**D**) at concentrations of 0.5 or 1.0 mM, from the L1 stage, for 72 h. The total number of animals examined was from 3 to 6 independent experiments performed on different days. Statistical differences between curves were determined using the Log-rank (Mantel–Cox) test [* *p* < 0.05 versus 0.0 mM (control)].

Drug abuse is associated with premature aging and a reduction in life expectancy. The mechanisms involved include a combination of direct toxic effects on various organs and biological systems, an increased risk of infectious diseases, generally poor health behaviors, and a higher risk of fatal overdoses [49]. Considering this information, we next evaluated the influence of exposing synchronized L1-stage animals to synthetic cathinones at 0.5 mM and 1.0 mM, for 72 h, on their longevity (Figure 6A). Exposure to methylone or pentedrone, at either 0.5 mM or 1.0 mM, did not significantly alter the animal’s lifespan, as determined by the Log-rank (Mantel–Cox) test [methylone: 0.5 mM, *p* = 0.543; 1.0 mM, *p* = 0.210; versus 0.0 mM (control); pentedrone: 0.5 mM, *p* = 0.412; 1.0 mM, *p* = 0.424; versus 0.0 mM (control)] (Figure 6B,C). However, exposure to the sublethal concentration of 0.5 mM of 4-MEC significantly shortened the animal’s lifespan [0.5 mM, *p* = 0.048; 1.0 mM, *p* = 0.063; versus 0.0 mM (control)] (Figure 6D). These results indicated that exposure to 4-MEC triggered premature animal death.

## 4. Discussion

### 4.1. Importance of Elucidating the Toxic Effects of Synthetic Cathinones

Synthetic cathinones represent an emerging class of NPS that has gained particular attention in recent years, especially in recreational settings. These drugs are often falsely marketed as legal alternatives to other illicit drugs, such as methamphetamine and MDMA. However, the increasing prevalence of synthetic cathinone use raises significant concerns about their toxic effects and impact on public health [3,25,50]. Data from synthetic cathinone users and non-fatal intoxication cases have shown a wide range of acute adverse effects, including tachycardia, hypertension, agitation, seizures, and chronic psychiatric, neurological, and cardiovascular changes [3,25].

Studying the toxic potential of methylone, pentedrone, and 4-MEC, even though they are not the most recent synthetic cathinones, is important for several reasons: (1) although they are among the first synthetic cathinones to gain popularity in the illicit drug market, significant gaps remain in understanding the potentially toxic effects of this class of drugs, especially in vivo; (2) the elucidation of their toxic effects can provide important information about the toxicity of synthetic cathinones in general, helping to predict and compare the toxic effects of newer derivatives that may share similar structural properties; (3) these substances might still be present in the drug market in regions where newer synthetic cathinones are less prevalent. Thus, even though new compounds continue to emerge in the illicit drug market [51,52], understanding the real toxic potential of older synthetic cathinones remains important.

In this study, exposure to the synthetic cathinones methylone, pentedrone, and 4-MEC for short- (24 h) or long-term (72 h) periods resulted in a marked reduction in animal survival (Figure 2C–E and Figure 3B–D; Tables S1 and S2). However, a more pronounced effect was observed after long-term exposure. Considering that the drugs were not replenished during the long-term incubation period, this result suggests that they likely persisted in the medium over time, allowing for continued exposure. Therefore, a single dose of synthetic cathinones may lead to prolonged effects of unpredictable magnitude. In vitro studies have reported pronounced toxic effects for these synthetic cathinones [14,15,16,17,18,19,20,53]. Additionally, several fatal intoxications involving these substances have been reported [54,55,56,57,58], highlighting their significant toxic potential. Notably, in a fatal intoxication involving 4-MEC, concentrations as high as 43.4 μg/mL (corresponding to 0.23 mM) and 619 μg/mL (corresponding to 3.24 mM) were detected in a postmortem analysis of cardiac blood and urine, respectively [55]. Considering that concentrations of 2.5 mM or higher reduced animal survival rates to nearly 0% for 4-MEC, and that pronounced effects on animal development were already observed at a sublethal concentration of 0.5 mM, these results support the range of concentrations used in our study. Additionally, they validate *C. elegans* as an interesting animal model for studying the potential toxicity of synthetic cathinones to humans.

Many synthetic cathinone users consume these substances continuously over several days, increasing the risk of cumulative toxicity [10,59]. Thus, the results of this study also highlight the need to inform consumers about the risks of continuous synthetic cathinone use.

### 4.2. Implications of Synthetic Cathinones’ Effects on Animal Development, Reproductive Behavior, and Lifespan

The increasing use of drugs of abuse by adolescents and young adults is a growing public health concern. One of the primary factors contributing to the rise in drug abuse among young people is the accessibility and social acceptance of certain substances. The prevalent use of social media and the internet has made drug-related information easier to access, and the combination of friend pressure and the impulse to either agree or resist can lead adolescents to try drugs [60]. The consequences of drug abuse in this age group are particularly severe. Adolescents and young adults are in a critical phase of brain and body development, and the use of psychoactive substances can interfere with the normal maturation process. This can result in long-term cognitive deficits, including impaired memory and reduced learning capacity. Moreover, drug use can exacerbate mental health issues such as depression, anxiety, and psychosis, which are already prevalent in this age group [36,37].

On the other hand, in humans, exposure to drugs of abuse during the fetal period is an additional concern. These substances have the potential to directly interfere with critical developmental stages or impair proper nutrient and energy intake, likely resulting in micronutrient deficiencies [38,39]. In fact, psychoactive substances like ethanol, cannabinoids, and tobacco have been associated with developmental abnormalities [39,61,62].

The developmental arrest observed in animals exposed to sublethal concentrations of 0.5 mM and 1.0 mM of synthetic cathinones (Figure 4B–G; Table S3) can indicate potential disruptions in essential biological pathways. This suggests that synthetic cathinones may have severe adverse effects on growth and development when exposure occurs during critical periods. Such interferences can have serious short- and long-term consequences for the organism’s functionality, resulting in poor health. These findings highlight the need for further research to understand the mechanisms underlying these effects and to evaluate the potential risks of synthetic cathinone exposure in humans, especially during developmentally sensitive periods such as adolescence or pregnancy.

Despite the potential of psychoactive substances to interfere with the normal development process, drug abuse can also significantly affect reproductive health [63]. The impact of drugs of abuse on reproductive systems varies depending on the substance and duration of use, affecting both males and females. Common effects include hormonal imbalances, reduced fertility, and complications during pregnancy [39,64].

This study also demonstrated that pentedrone (1.0 mM) reduced the *C. elegans* reproductive capacity (Figure 5C; Table S4). Therefore, a detailed characterization of the potential interference of synthetic cathinones—and, by extension, other drugs of abuse—at this level represents an area of growing interest in the current context, with significant public health implications. We also showed that the exposure of animals to synthetic cathinones did not significantly affect the viability of their progeny (Figure 5E–G; Table S5). Nevertheless, the possibility of transgenerational effects cannot be ruled out, as adverse effects may emerge after repeated exposures over extended periods. Thus, further investigation is needed to assess whether prolonged or cumulative exposure to these substances could lead to detrimental effects in subsequent generations.

Additionally, the chronic use of drugs of abuse is often associated with premature aging and reduced longevity. This association arises from a combination of direct toxic effects on the cells and organs of the organism, as well as an increased risk of developing various diseases, including cardiovascular, liver, and neurological disorders. These damaging effects cumulatively contribute to an overall decline in health and accelerated aging processes [49]. Thus, the significant reduction in the lifespan of animals exposed to 4-MEC (Figure 6D) may indicate that this synthetic cathinone (and potentially other drugs in this class with increased toxicity) can trigger mechanisms leading to early onset aging. Additionally, the reduction in longevity observed in *C. elegans* suggests that similar biological mechanisms might occur in humans, where prolonged exposure to these substances could accelerate aging and increase susceptibility to serious health conditions. Thus, the mechanism(s) behind this effect warrant(s) further investigation.

Overall, the potential implications of these results for public health are significant, highlighting the need for further research and concerns regarding the long-term effects of synthetic cathinones on human health.

### 4.3. C. elegans as a Model for Toxicity Studies on Drugs of Abuse

To elucidate the toxic effects of synthetic cathinones, various studies have utilized in vitro cellular models [14,15,16,17,18,19,20,21,22]. Specifically, studies using neuronal [17,18,19,20], kidney [16], and hepatic [14,15] models have reported pronounced toxic effects for the synthetic cathinones investigated in this work. These studies have provided important insights into the mechanisms of toxicity of these substances and, by extension, synthetic cathinones in general. However, in vitro, two-dimensional cellular models consisting of a single cell type are simplistic systems that do not replicate the complex systemic interactions present in living organisms. In the human body, cells, tissues, and organs interact dynamically, mutually influencing their functions and responses to stimuli. In vitro, two-dimensional, single-cell models lack these interactions, which may lead to results that do not accurately reflect the biological behavior observed in vivo [65].

In this study, the use of *C. elegans* as an animal model not only overcomes the main limitations of two-dimensional in vitro cellular systems but also offers numerous additional advantages. They include the ease of manipulation in the laboratory and the organism’s safety (since it does not develop at temperatures above 25 °C, it does not pose a health risk to humans), rapid reproductive cycle, and short lifespan [48]. These characteristics make this model suitable for exploring the effects of substances on development, reproductive behavior, and longevity. Furthermore, the high sensitivity of this animal model to toxic agents and the ability to conduct large-scale toxicity studies efficiently and at low cost make it an ideal choice for preliminary investigations [29,40]. Additionally, the similarity to humans in certain aspects of cellular function and regulatory mechanisms provides significant advantages, allowing for some degree of extrapolation of the results obtained in this model to human health [32]. However, to date, only a few studies have utilized this animal model to elucidate the toxic effects of drugs of abuse, specifically amphetamine [66,67,68,69] and piperazine [70] derivatives, cocaine [71], ethanol [72,73,74,75,76], as well as ketamine [77], an anesthetic drug that has gained increased notoriety as a drug of abuse in contemporary society [3]. Nevertheless, as research on the effects of drugs of abuse continues to grow, *C. elegans* will likely play a crucial role in advancing our understanding of the toxicity (and associated mechanisms) of these substances, as well as its implications for public health.

### 4.4. Study Limitations and Future Perspectives

Although this study provides important insights into the toxic potential of the synthetic cathinones methylone, pentedrone, and 4-MEC, and, by extension, the class of synthetic cathinones in general, it also has some limitations. Firstly, the fact that toxicity studies were conducted under controlled laboratory conditions may not fully reflect the real scenarios of the use of these substances. Synthetic cathinones are commonly used in recreational environments such as nightclubs, bars, or festivals, which are typically overheated places [3,13]. Considering that hyperthermia is one of the most characteristic toxic effects of these substances and significantly contributes to their overall toxicity [10,78], the intake of such drugs in these contexts may exacerbate hyperthermia-associated toxic effects. However, the *C. elegans* animal model does not develop at temperatures above 25 °C [30,40]. While this is advantageous because its use does not pose any risk to human health, it also limits the laboratory replication of the hyperthermic conditions typically associated with the environments in which these substances are consumed.

Moreover, differentiated aspects in the ADME process, such as variations in absorption (due to variations in the composition and organization of physiological barriers), distribution, metabolic pathways/enzymatic systems, and excretion between *C. elegans* and humans, may represent additional obstacles in extrapolating these results to humans. Thus, the use of a single animal model may not fully elucidate the toxic effects triggered by these substances in more complex organisms, such as humans. Therefore, complementary studies in other animal models, including mammals, can help in depicting a more realistic and comprehensive understanding of the toxicological effects of xenobiotics and the mechanisms involved. By integrating data from multiple models, researchers can better account for interspecies differences and improve the accuracy of predictions related to human health risks.

## 5. Conclusions

This study highlights the toxicological effects of synthetic cathinones methylone, pentedrone, and 4-MEC, using the model organism *C. elegans*. Short-term exposure (24 h) to these drugs caused a concentration-dependent decrease in animal survival, with prolonged exposure exacerbating the toxicity. At sublethal concentrations, synthetic cathinones delayed animal development. Additionally, pentedrone reduced reproductive performance, while 4-MEC shortened animal longevity.

This represents the first study to evaluate, in vivo, the toxic effects of synthetic cathinones in the *C. elegans* animal model. These findings underscore the serious toxic effects of synthetic cathinones, which are likely to be relevant in other biological systems, including humans. As synthetic cathinones continue to rise in the illicit drug market, this study emphasizes the urgent need for further research using in vivo models to fully understand their impact on human health. Additionally, rigorous regulation, public awareness campaigns to educate the public about the risks, and advanced monitoring to track emerging drug trends are essential to help prevent the abuse of synthetic cathinones.

## Data Availability

The original contributions presented in this study are included in the article/Supplementary Materials. Further inquiries can be directed to the corresponding author.

## Supplementary Materials

The following supporting information can be downloaded at: https://www.mdpi.com/article/10.3390/jox15010033/s1, Figure S1: 400 MHz one-dimensional (1D) ^1^H-Nuclear Magnetic Resonance (^1^H-NMR) spectra of methylone, pentedrone, and 4-MEC in deuterium oxide (D_2_O) with 1 mM 3-(trimethylsilyl)propionic-2,2,3,3-d4 acid (TSP); Table S1: Short-term exposure (24 h) to the synthetic cathinones methylone, pentedrone, and 4-MEC led to a concentration-dependent reduction in animal survival; Table S2: Long-term exposure (72 h) to the synthetic cathinones methylone, pentedrone, and 4-MEC led to a concentration-dependent reduction in animal survival; Table S3: Exposure to sublethal concentrations of synthetic cathinones for 72 h arrested animal development; Table S4: Exposure to a sublethal concentration of 1.0 mM pentedrone for 72 h reduced the *C. elegans* brood size; Table S5: Exposure of animals to sublethal concentrations of synthetic cathinones for 72 h did not affect the viability of their progeny.

## Abbreviations

The following abbreviations are used in this manuscript:

1D ^1^H-NMR	One-dimensional ^1^H-Nuclear Magnetic Resonance
4-CMC	4-Chloromethcathinone
4-FMC	4-Fluoromethcathinone
4-MEC	4-Methylethcathinone
4-MMC	4-Methylmethcathinone
CMOS	Complementary Metal Oxide Semiconductor
CNS	Central Nervous System
DAT	Dopamine Reuptake Transporter
EUDA	European Union Drugs Agency
MDMA	3,4-methylenedioxymethamphetamine
MDPV	3,4-methylenedioxypyrovalerone
NAT	Noradrenaline Reuptake Transporter
NGM	Nematode Growth Medium
NPS	New Psychoactive Substances
SEM	Standard Error of the Mean
SUD	Substance use disorder
TSP	3-(trimethylsilyl)propionic-2,2,3,3-d4 acid
