# Supplementary material for: Synthetic Cathinones Induce Developmental Arrest, Reduce Reproductive Capacity, and Shorten Lifespan in the C. elegans Model
Source: J Xenobiot. 2025 Feb 18;15(1):33. doi: 10.3390/jox15010033 (PMC11856764; doi:10.3390/jox15010033)
Supplement: Supplementary file 1 [file jox-15-00033-s001.zip › jox-3451041-supplementary.pdf]

Supplementary materials

Synthetic cathinones induce developmental arrest, reduce reproductive capacity, and shorten lifespan in the *C. elegans* model

Cristina Mendes [1,2], Daniela Maia [1,2], Ricardo Jorge Dinis-Oliveira [1,2,3,4], Fernando Remião [5,6], Renata Silva [5,6] and Daniel José Barbosa [1,2,7,*]

1 Associate Laboratory i4HB—Institute for Health and Bioeconomy, University Institute of Health Sciences—CESPU, 4585-116 Gandra, Portugal; a33272@alunos.cespu.pt (C.M.); danielamaia26@outlook.pt (D.M.); ricardo.dinis@iucs.cespu.pt (R.J.D.-O.)

2 UCIBIO—Applied Molecular Biosciences Unit, Translational Toxicology Research Laboratory, University Institute of Health Sciences (1H-TOXRUN, IUCS-CESPU), 4585-116 Gandra, Portugal

3 Department of Public Health and Forensic Sciences and Medical Education, Faculty of Medicine, University of Porto, 4200-319 Porto, Portugal

4 FOREN—Forensic Science Experts, Dr. Mário Moutinho Avenue, No. 33-A, 1400-136 Lisbon, Portugal

5 Associate Laboratory i4HB—Institute for Health and Bioeconomy, Faculty of Pharmacy, University of Porto, 4050-313 Porto, Portugal; remiao@ff.up.pt (F.R.); rsilva@ff.up.pt (R.S.)

6 UCIBIO—Applied Molecular Biosciences Unit, Laboratory of Toxicology, Department of Biological Sciences, Faculty of Pharmacy, Porto University, 4050-313 Porto, Portugal

7 i3S—Instituto de Investigação e Inovação em Saúde, Universidade do Porto, 4200-135 Porto, Portugal

* Correspondence: daniel.barbosa@iucs.cespu.pt

Supplementary Materials

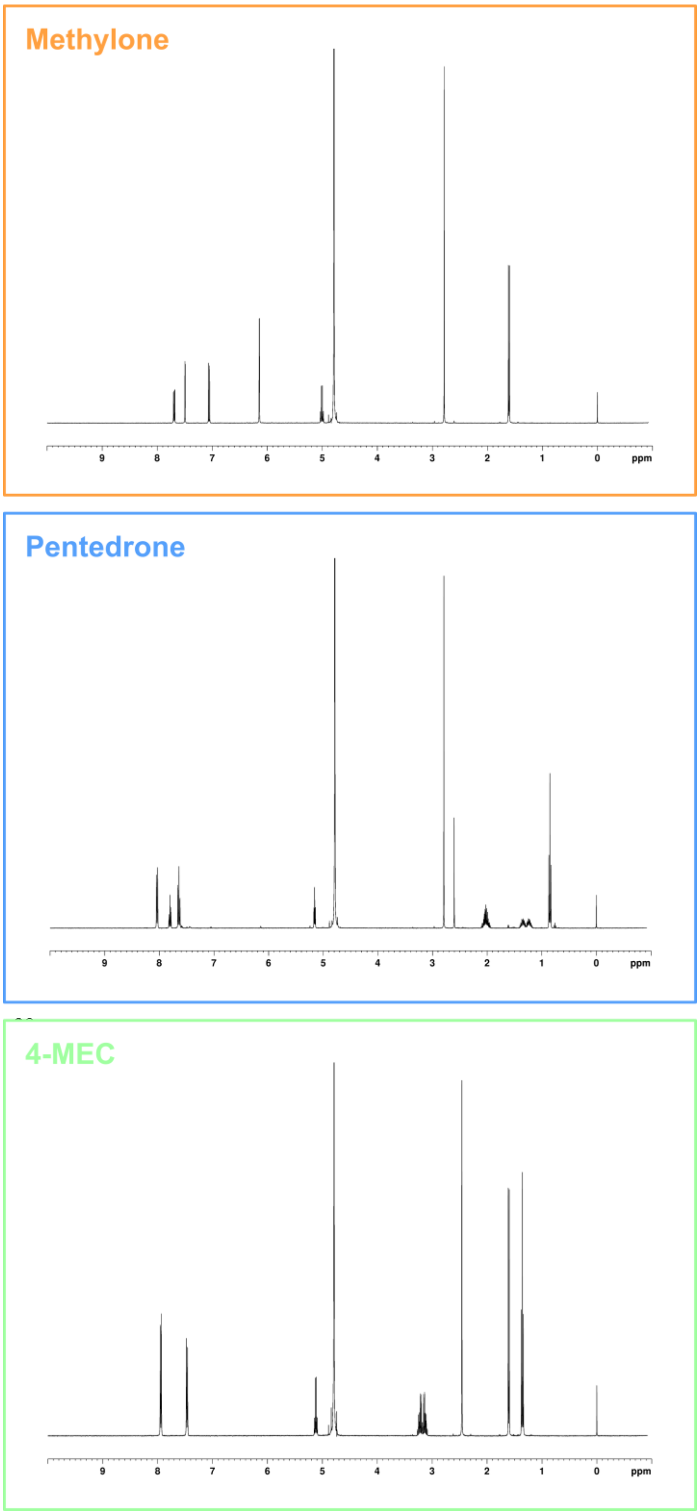

Figure S1. 400 MHz one-dimensional (1D) ^{1}H-Nuclear Magnetic Resonance (^{1}H-NMR) spectra of methylone, pentedrone, and 4-MEC in deuterium oxide (D_2O) with 1 mM 3-(trimethylsilyl)propionic-2,2,3,3-d4 acid (TSP).

Table S1. Short-term exposure (24 h) to the synthetic cathinones methylone, pentedrone, and 4-MEC led to a concentration-dependent reduction in animal survival.

	Concentration (mM)						
	**0.0**	**0.1**	**0.5**	**1.0**	**5.0**	**7.5**	**10.0**
**Methylone**	90.9 ± 2.2%	85.4 ± 3.2%	82.1 ± 3.5%	82.2 ± 2.9%	67.4 ± 4.1%*	30.9 ± 4.6%***	20.3 ± 4.5%****
**Pentedrone**	92.6 ± 2.7%	89.4 ± 3.2%	84.5 ± 4.1%	82.6 ± 3.2%	54.3 ± 7.2%**	18.0 ± 3.3%**	1.7 ± 1.0%****
**4-MEC**	89.8 ± 2.7%	87.7 ± 3.8%	84.6 ± 3.8%	83.4 ± 4.1%	48.9 ± 10.6%*	20.8 ± 4.9%**	13.3 ± 3.6%***

Results represent the mean ± SEM of the percentage of surviving animals from 5 to 7 independent experiments performed on different days. Statistical differences were determined by the Krus-kal-Wallis nonparametric test, followed by Dunn's multiple comparison test. [*$p < 0.05$; **$p < 0.01$; ***$p < 0.001$; ****$p < 0.0001$ versus 0.0 mM (control)].

Table S2. Long-term exposure (72 h) to the synthetic cathinones methylone, pentedrone, and 4-MEC led to a concentration-dependent reduction in animal survival.

	Concentration (mM)				
	**0.0**	**0.5**	**1.0**	**2.5**	**5**
**Methylone**	96.4 ± 0.9%	91.0 ± 2.0%	86.8 ± 2.1%	66.6 ± 5.8%***	8.2 ± 4.2%****
**Pentedrone**	96.6 ± 1.1%	92.2 ± 0.8%	77.8 ± 4.3%*	15.2 ± 5.5%****	0.6 ± 0.6%****
**4-MEC**	95.4 ± 1.8%	92.0 ± 2.1%	54.5 ± 10.3%*	0.3 ± 0.2%***	0.0 ± 0.0%***

Results represent the mean ± SEM of the percentage of surviving animals from 10 to 11 independent experiments performed on different days. Statistical differences were determined by the Krus-kal-Wallis nonparametric test, followed by Dunn's multiple comparison test. [*$p < 0.05$; ***$p < 0.001$; ****$p < 0.0001$ versus 0.0 mM (control)].

Table S3. Exposure to sublethal concentrations of synthetic cathinones for 72 h arrested animal development.

	Concentration (mM)		
	**0.0**	**0.5**	**1.0**
**Methylone**	100.0 ± 1.8%	99.4 ± 2.6%	77.6 ± 2.1%****
**Pentedrone**	100.0 ± 2.6%	74.6 ± 2.2%****	61.1 ± 1.6%****
**4-MEC**	100.0 ± 1.8%	84.0 ± 1.9%****	60.0 ± 1.5%****

Animal length (% of control) is represented as the mean ± SEM from 4 to 5 independent experiments performed on different days. Statistical differences were determined by the Kruskal-Wallis nonparametric test, followed by Dunn's multiple comparison test [****$p < 0.0001$ versus 0.0 mM (control)].

Table S4. Exposure to a sublethal concentration of 1.0 mM pentedrone for 72 h reduced the *C. elegans* brood size.

	Concentration (mM)		
	**0.0**	**0.5**	**1.0**
Methylone	139.2 ± 6.5	126.6 ± 6.1	120.6 ± 6.8
Pentedrone	136.2 ± 6.9	125.5 ± 6.1	113.8 ± 5.6*
4-MEC	160.7 ± 7.2	150.1 ± 5.9	141.0 ± 9.8

Data represent the mean ± SEM of the total progeny of F0 animals, assessed in 3 to 6 independent experiments performed on different days. Statistical differences were determined by the Kruskal-Wallis nonparametric test, followed by Dunn's multiple comparison test [*$p < 0.05$ versus 0.0 mM (control)].

Table S5. Exposure of animals to sublethal concentrations of synthetic cathinones for 72 h did not affect the viability of their progeny.

	Concentration (mM)		
	**0.0**	**0.5**	**1.0**
Methylone	96.7 ± 1.6%	97.0 ± 1.2%	97.1 ± 1.1%
Pentedrone	96.7 ± 1.0%	96.4 ± 1.6%	97.1 ± 1.2%
4-MEC	98.3 ± 0.5%	98.3 ± 0.4%	96.9 ± 1.0%

Data represent the mean ± SEM of the percentage of hatched embryos (F1 generation), assessed in 3 to 7 independent experiments performed on different days. Statistical analysis was performed using the Kruskal-Wallis nonparametric test.
